# A Follow-Up Study on the Influence of Personal, Family, and School Factors on Learning Outcomes of Students with Disabilities in Senior High School

**DOI:** 10.3390/bs13070554

**Published:** 2023-07-04

**Authors:** Shu-Jou Sun, Wei-Sho Ho

**Affiliations:** 1 Department of Special Education, National Tsing Hua University, No. 521, Nanda Rd., East Dist., Hsinchu City 300193, Taiwan; sjsun@mx.nthu.edu.tw; 2 Department of Industrial Education and Technology, National Changhua University of Education Bao-Shan Campus, No.2, Shi-Da Rd., Changhua City 500208, Taiwan; 3 NCUE Alumni Association, National Changhua University of Education Jin-De Campus, No. 1, Jinde Rd., Changhua City 500207, Taiwan

**Keywords:** special needs education longitudinal study, secondary analysis, students with disabilities, learning outcomes

## Abstract

The purposes of this study were to describe the learning outcomes of students with disabilities in senior high school, to establish a model to explain the effects of personal, family, and school experience factors on the learning outcomes of students with disabilities, and to determine the relationship between post-school and in-school outcomes. There were 496 participants selected in the 2011 and 2012 academic year from the database of Special Needs Education Longitudinal Study. The survey data obtained from questionnaires for teachers, parents, and students were used to conduct secondary analysis. Descriptive statistics such as frequencies and percentages, a PLS structural equation model, and multiple regression were used in this study. The results of this study were as follows: (1) Students with disabilities had the best learning performances in school, and most parents were satisfied with their students’ education in school; however, employment performance was the weakest upon leaving school. (2) School experience factors had the greatest influence on the school learning outcomes model, followed by student factors and family factors. (3) In-school outcomes effectively predicted postsecondary education, employment, social adaptation, and satisfaction after leaving school. In conclusion, the results of this study found that personal, family, and school factors have a significant impact on the learning outcomes of students with disabilities, and in-school outcomes can effectively predict postsecondary education, social adaptation, and satisfaction after leaving school.

## 1. Introduction

Since the announcement of the Education for All Handicapped Children Act in 1975 in the United States, it has been emphasized that the federal government should understand the effectiveness of special education implemented by state and local educational institutions through evaluation [1,2]. This act was amended in 1990 as The Individuals with Disabilities Education Act (IDEA). Article 612 emphasizes that states should establish performance indicators for students with disabilities to measure their progress [3,4]. It was revised again in 2004, emphasizing that all students with disabilities, like general students, must receive regular state-wide or school-district assessments to understand their learning outcomes in general education subjects [5]. In Taiwan, Article 47 of the Special Education Law also stipulates: “The effectiveness of special education in schools at all levels of education below senior high school shall be evaluated by the competent authority at least every four years” to understand the accountability of special education. 

Students with disabilities have higher rates of absenteeism and dropouts [6,7], their academic performance lags behind that of general students [7,8,9], and their graduation rate is relatively low [6], but there is no obvious difficulty in social adaptation and self-care ability [8]. Furthermore, the rate of students with disabilities participating in postsecondary education and employment increases as the time of leaving school increases [7,10]. Conversely, the frequency of community volunteer service and participation in club organization activities decreases with an increase in the time away from school [7,11]. 

It can be seen that the special education regulations of the United States and Taiwan both emphasize the learning outcomes of students with disabilities and the effectiveness of special education through regular assessments. However, there few studies on the learning outcomes of students with disabilities in high school in Taiwan. Therefore, one of the purposes of this study is to explore the learning outcomes of students with disabilities in senior high school.

Secondly, there are many factors related to learning outcomes. Many studies have found that the characteristics of students’ disabilities, gender, intelligence, school environment, and school size will affect students’ learning outcomes [6,12,13,14,15]. Wagner et al. (2003) also pointed out that personal background variables such as student disability categories, gender, and race; family background variables such as family income, parental participation, and parental expectations; and school background variables such as time spent participating in regular class activities, receiving teaching adjustments, and support services were important factors affecting learning outcomes. Therefore, the second purpose of this study is to construct an impact model of learning outcomes of students with disabilities [8].

## 2. Literature Review

### 2.1. Learning Outcomes

Learning outcomes refer to the results that students acquire after receiving education in school, including cognitive learning, emotional expression, psychosocial development, practical ability, values, attitudes, skills, etc. [16,17,18,19]. In the field of education, students’ learning outcomes are mostly informed by their academic achievements, specifically their mastery of subject content and the school’s accountability [8]. Second, attendance is the most basic indicator of participation in school activities, and high absenteeism is an important predictor of academic failure and dropout among students with disabilities [20]. This is because adolescents spend most of their time at school, and school is where they learn to solve problems, follow instructions, and build relationships with peers and adults. Therefore, adolescents’ behavior at school is also a key factor in social adjustment. 

For regular students and students with disabilities, the main purpose of education is to prepare for future adult life. Before the mid-1990s, most of the post-school outcomes of students with disabilities emphasized employment outcomes. However, studies have shown that before 1959, only 20% of workers required at least a college degree for their jobs, and this had increased to 56% by 2000 [21]. Therefore, being able to access and participate in postsecondary education is an important challenge in secondary education and transition for students with disabilities [22]. Secondly, the use of leisure time and the time spent with friends by regular students and students with disabilities after leaving school are also different from those at school, including the use of leisure time, interaction with friends, and participation in community organizations or activities [23].

The National Longitudinal Transition Study of Special Education Students (NLTS) proposes that academic achievement, graduation rate, postsecondary education, employment, and independence are the learning outcomes of middle school students in and out of school [7]. Since 2000, The National Longitudinal Transition Study-2 (NLTS2) has proposed four dimensions of school participation, academic performance, social adaptation, and independence as in-school outcomes, and postsecondary education, employment, independence, social participation, and civic activities have been proposed post-school outcomes [8,23]. In Taiwan, the SNELS also divides the educational outcomes of students with disabilities into two parts: in-school outcomes and post-school outcomes. In-school outcomes include learning participation, learning performance, life and independence, social adaptation, satisfaction, and family outcomes, while post-school outcomes include employment and vocational rehabilitation, postsecondary education, responsibility and independence, social adaptation, self-determination, health and safety, and satisfaction.

### 2.2. In-School and Post-School Outcomes

Wagner et al. (1993) analyzed NLTS data and found that 18.7% of students were absent for more than 20 days per semester, and the dropout rate of students with emotional disabilities was the highest [7]. Wagner et al. (2003) analyzed the first wave of NLTS2 data and found that only 30% of students with disabilities fell between A and B in subject achievement and averaged 3.6 grades behind general students in reading and math tests. However, most of the students got along well with their peers and had good self-care skills [8]. Wagner et al. (2006a) analyzed the second wave of NLTS2 data and also found that 14–27% of students with disabilities had a score of less than 70 on the standardized achievement test, among which reading comprehension was scored the lowest [9]. Barrat et al. (2014) compared the results of students with disabilities and regular students in grades 6–12 in Utah, showing that the dropout rates of students with disabilities were higher than those of regular students, and the graduation rate were lower than that of regular students [6].

It can be seen that in terms of in school learning outcomes, students with disabilities have higher absentee and dropout rates, poor academic performance, and lower graduation rates than regular students, but exhibit better in social adaptation and independence [6,7,8,9,24,25]. 

Wagner et al. (2005) analyzed the results of students with disabilities within two years of leaving school and found that 30.6% of students participated in postsecondary education, with most attending two-year community colleges and the least attending four-year universities [23]. Additionally, 42.9% of students participated in employment, and only 28% of them joined social organizations. Wagner et al. (1993) compared the outcomes of students with disabilities who had left school for two years and those who had left school for three to five years and found that the participation rate in postsecondary education increased from 14% to 26.7% [7] and the rate of participation in competitive employment increased from 45.7% to 57.8%. However, their weekly interaction rate with friends or other family members decreased from 51.9% to 38.2%, and the rate of participation in club activities also decreased from 28.0% to 21.4%. In addition, Newman et al. (2011) compared the results of students with disabilities within three years after leaving school and within five to eight years and found that the rate of participation in postsecondary education increased from 52.3% to 61.9%, the rate of participation in employment increased from 49.5% to 59.1% and the rate of joining social organizations increased from 30.2% to 42.2% [26].

Based on the above discussion, we know that the participation rate of students with disabilities in postsecondary education and employment will increase with an increase of time away from school. After leaving school, two-year community colleges are the most common, and four-year universities are the least common. Also, the rate of interacting with friends is significantly lower [7,23]. However, studies have found that the rate of students with disabilities participating in social organizations and activities after graduation produces inconsistent results [7,26]. Therefore, this study will explore the outcomes of high school students with disabilities leaving school from the perspectives of postsecondary education, employment, social adaptation, and satisfaction.

In Taiwan, the Special Needs Education Longitudinal Study (SNELS) is a longitudinal database which was established to collect the data of individuals with disabilities, their families, and schools in the four stages of education: preschool, primary school, junior high school, and senior high school. This is done in a comprehensive and longitudinal manner, facilitating investigations into the important issues for the education of individuals with disabilities. It also allows for related analysis of the teaching situation and educational achievements of students with disabilities [27,28].

Therefore, the main purpose of this study was to use the data from the SNELS to explore the learning outcomes of students with disabilities in senior high school, the influence model of the learning outcomes of students with disabilities, and the correlation between post-school and in-school outcomes. The research questions are as follows: (1) According to the data of the 2011 and 2012 academic years, what are the learning outcomes of students with disabilities in-school and post-school? (2) What are the influences of personal factors, family factors, and school experience factors on learning outcomes of students with disabilities in senior high school? (3) According to the data of the 2011 and 2012 academic years, which dimensions of the in-school learning outcomes can effectively predict post-school postsecondary education, employment, social adaptation, and satisfaction?

## 3. Methodology

### 3.1. Research Model

**Figure 1:**
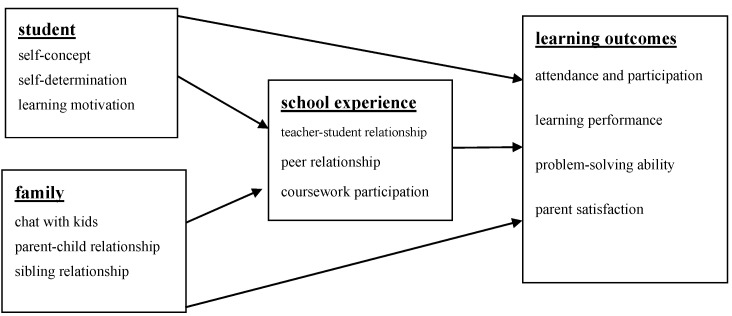
Learning outcomes impact model for students with disabilities.

The researchers refered to the three databases, NLTS [7], NLTS2 [8], and SNELS, to define the scope of in-school outcomes, and IDEA emphasizes the rights of parents to participate in the learning activities of students with disabilities and to review learning outcomes. Therefore, the researchers constructed a theoretical framework for the learning outcomes of students with disabilities in school, and structure of this study is shown in Figure 1. The latent dependent variable was learning outcomes, including attendance and activity participation, learning performance, problem-solving ability, and parent satisfaction. Latent independent variables included student factors, family factors, and school experience factors. Therefore, this study used school experience as the mediating variable to construct an impact model on the learning outcomes of students with disabilities in high school.

### 3.2. Participants

The participants in this study consisted of the data from the 2011 and 2012 academic years released by the SNELS. From the 2011 academic year data file, the researchers found that there were a total of 3654 students with disabilities in the first and third years of senior high school. After excluding those who left after the first year of senior high school, 1451 students remained, and 496 students were selected and placed exclusively in general classes. The total number of samples released in the 2012 academic year was 1293. However, since the placement category of the sample in the third year of senior high school was unknown, the researchers selected the subjects who were placed in the regular class in the 2011 academic year with the same student code. Due to data loss for 92 participants, a total of 404 students who had left school were obtained. The 496 participants of this study in the 2011 academic year and their areas of residence, gender, and disability categories are shown in Table 1. Table 1 shows that the largest number of students with disabilities lived in the northern area (25.0%), followed by the central area (24.4%). There were more males than females (64.7%), and the most common types of disabilities were orthopedic disabilities (17.7%), followed by learning disabilities (16.9%).

### 3.3. Measures

In this study, certain topics were selected as observed indicators from the student questionnaire, teacher questionnaire, and parent questionnaire in the 2011 academic year, and the student questionnaire and parent questionnaire in the 2012 academic year (there was no teacher questionnaire because they had left school). Secondly, the researchers conducted factor analysis on the selected topics, and the names of the factors are shown in Table 2 and Table 3. There were four options for the selected questions, and the scoring method was given from one to four points. Table 2 shows the latent independent variables, including students, families, and schools. Table 3 shows the latent dependent variables, including in-school and post-school outcomes.

### 3.4. Data Analysis

This study used data from the SNELS database for secondary data analysis. Firstly, the researchers used the frequency distribution and percentage to understand the learning outcomes of students with disabilities in and out of school to answer research question 1. The PLS-SEM structural equation model was used to verify the impact model of learning outcomes of students with disabilities constructed in this study to answer research question 2. Secondly, the relationship between in and out of school outcomes was analyzed using a Pearson product–moment correlation, followed by multiple regression analysisto predict the in-school outcomes and post-school outcomes to answer research question 3.

## 4. Results

### 4.1. In-School and Post-School Learning Outcomes of Students with Disabilities

The learning outcomes of students with disabilities in school are shown in Table 4. The results show that the absentee rate of students with disabilities was relatively high, with a sometimes absentee rate of 52.4% and a frequent absentee rate of 1.4%. In terms of learning performance, including listening attentively in class, following teacher instructions, completing homework on time, and being able to focus on learning tasks, the agreement rate was as high as 80%. In terms of problem-solving ability, including making appropriate choices and decisions by themselves, finding solutions when encountering difficulties, managing their own time, and knowing the direction of their future career development, nearly 20% of the disabled students disagreed, especially for their future career development planning, and as many as 33.9% of students with disabilities had no clear direction. 

As far as parent satisfaction was concerned, although more than 80% of parents were satisfied with their children’s relationships with teachers, classmates, and participation in school activities, nearly 17% of parents were still dissatisfied with their children’s learning progress at school. The results show that students with disabilities in the third year of senior high school had better school outcomes in terms of “learning outcomes”, and nearly 20% of the students said that they had difficulties in “problem-solving ability”. Most parents were satisfied with the overall situation of students with disabilities receiving education in school. 

This study refers to relevant literature and lists the four dimensions of postsecondary education, employment, social adaptation, and satisfaction as the learning outcomes of school-leaving students. Table 5 shows the learning outcomes of school-leaving students with disabilities in the 2012 academic year. Among the 404 students with disabilities who left school, only 315 continued to enter universities after graduation (78.0%), and only 43 were employed (10.6%). 

In terms of school-leaving outcomes, students with disabilities performed better in postsecondary education, social adaptation, and satisfaction, and performed poorly in employment. In particular, the employment rate of the participants in this study was extremely low. Only 8.9% of participants were satisfied with the salary of their current job, and only 9.7% liked their current job. Secondly, although the students with disabilities who had left school were more than 90% satisfied with their current living environment and conditions, more than 17% of parents were still dissatisfied with the current living conditions of their children with disabilities.

### 4.2. The Learning Outcomes Impact Model

The researchers used PLS-SEM to develop the impact model of the learning outcomes and found that the factor loading of “attendance and participation” was too low, so it was deleted. Based on the results, the factor loadings of individual variables ranged from 0.543 to 0.870, and from the perspective of compositional reliability, they ranged from 0.749 to 0.814. The average variation extraction of latent variables ranged from 0.505 to 0.597, conforming to the value suggested by Fornell and Larcker (1981) [29]. 

**Figure 2:**
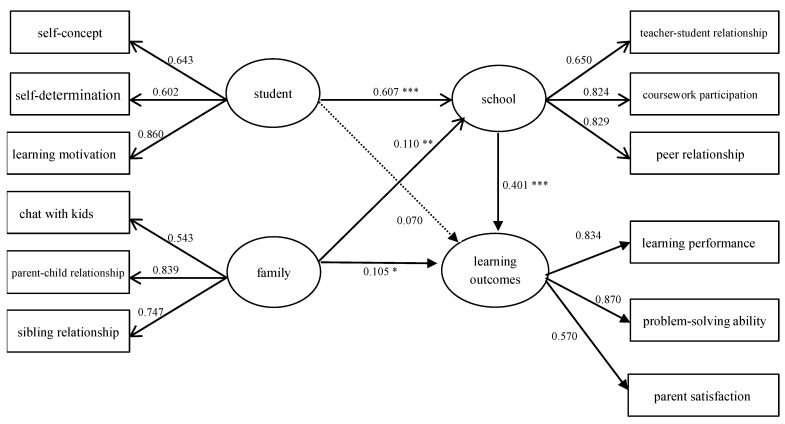
Learning outcomes model for students with disabilities. * *p* < 0.05 ** *p* < 0.01 *** *p* < 0.001.

Secondly, based on the path relationship of the pattern of influencing factors on the learning outcomes of students with disabilities shown in Figure 2, the direct effect of individual student factors on learning outcomes did not reach the significance level of 0.05 (t < 1.96). The rest of the path relationships reached significant levels above 0.05 (t > 1.96). It can be seen that “student factors” had a greater impact on “school experience”, with a path coefficient of 0.61 (t > 3.29, *p* < 0.000). “School experience” also had a significant positive and direct impact on “learning outcomes”, with a path coefficient of 0.40 (t > 3.29, *p* < 0.000), and “student factors” and “family factors” also had a positive indirect impact on “learning outcomes” through “school experience”. The results also show that “school experience”, “student factors”, and “family factors” are all important influencing factors for the learning outcomes of students with disabilities. In terms of explanatory power, “student factors”, “family factors”, and “school experience” can explain 24.3% of the variance in the learning outcomes of students with disabilities, of which 16.1% of the variance comes from “school experience” and 8.2% of the variance comes from student and family factors. In conclusion, among the impact modes of the learning outcomes of students with disabilities, “school experience” had the greatest influence, followed by student factors and family factors.

### 4.3. Prediction of In-School Outcomes on Post-School Outcomes

Based on the impact model of the learning outcomes of students with disabilities shown in Figure 2, the researchers first conducted a product–moment correlation analysis between the three dimensions of in-school outcomes, including: learning performance, problem-solving ability, parent satisfaction, and the four dimensions of school-leaving outcomes, including: postsecondary education, employment, social adaptation, and satisfaction. According to the correlation analysis, although the correlation between most dimensions was significant, the correlation coefficients were all below 0.3, which indicates a low correlation. Next, the researchers conducted a stepwise regression analysis using the three dimensions of in-school outcomes as independent variables and the four dimensions of school-leaving outcomes as dependent variables. The results are shown in Table 6, Table 7, Table 8 and Table 9.

The results in Table 6 show that among the in-school outcomes of students with disabilities, there were two variables that could effectively predict postsecondary education after leaving school. They were parent satisfaction and learning performance (parent satisfaction *β* = 0.204, *p* < 0.000, learning performance *β* = 0.150, *p* = 0.002) which could explain 7.6% of the variance in total. Among them, the amount of variation that could be explained by parent satisfaction was relatively high. Secondly the results in Table 7 show that among the in-school outcomes of students with disabilities, only the learning performance could effectively predict employment after leaving school (*β* = −0.155, *p* = 0.002), but it could only explain 2.4% of the variance. The results in Table 8 show that among the in-school outcomes of students with disabilities, there were two variables that could effectively predict the social adaptation after leaving school. They were parent satisfaction and problem-solving ability (parent satisfaction *β* = 0.184, *p* < 0.000, problem-solving ability *β* = 0.133, *p* = 0.007) which could explain 6.1% of the variance, of which parent satisfaction could explain a higher amount of the variation. The results in Table 9 show that among the in-school outcomes of students with disabilities, only parent satisfaction could effectively predict satisfaction after leaving school (*β* = 0.363, *p* < 0.000), which could explain 13.2% of the variance.

## 5. Discussion

The results of this study demonstrated that the learning outcomes of students with disabilities were better in “learning performance”, and the students experienced more difficulties in “problem-solving ability”. Most students could participate in school activities but had a high absentee rate, and parents were satisfied with the education situation of students with disabilities in schools. This result is consistent with Wagner et al. (1993) and Wagner et al. (2003), who found that the absentee rate of students with disabilities is higher than that of general students [7,8]. Secondly, Newman (2005) analyzed the Special Education Elementary Longitudinal Study (SEELS) and NLTS2 database and pointed out that more than 85% of parents are satisfied with the overall situation of school education for their children with disabilities [30], which is also consistent with the findings of this study. Secondly, because the participants in this study were all placed in general classes, nearly 17% of parents were less satisfied with the learning progress of their children with disabilities. This situation is also consistent with Barrat et al. (2014), Newman et al. (2011), Paul (2011), and Wagner (1993), who found that students with disabilities performed worse than their peers [6,7,14,26].

In terms of the performance situation of school-leaving outcomes, students with disabilities performed better in postsecondary education, social adaptation, and satisfaction, and they performed the worst in employment. According to the analysis of the NLTS database, the participation rate of students with disabilities in postsecondary education within two years of leaving school was 14%, and the employment rate was as high as 46% [7]. The analysis of the second wave of the NLTS2 database also showed that the participation rate of students with disabilities in postsecondary education within two years of leaving school was 30.6%, and the employment rate was as high as 43%. This result is inconsistent with the findings of this study, which may be due to the fact that the participants in this study had only graduated from senior high school for one year, most of them chose to pursue higher education, and relatively few were employed [23]. 

Secondly, this study explored the impact model of the in-school learning outcomes and found that “family factors”, “student factors”, and “school experience” are all important factors. Among them, “school experience” has the greatest influence, followed by student factors and family factors. Dell’Anna et al. (2022) found that teachers provide more teaching time and increase opportunities for students with disabilities to interact with general peers, which can improve their learning outcomes [31]. Doren et al. (2012) also found that student factors and parent expectations have a significant relationship with children’s learning outcomes [32], which are consistent with the results of this study. In addition, this study also explored the prediction results of students with disabilities in school on school leaving outcomes, although in-school outcomes could effectively predict postsecondary education, employment, social adaptation, and satisfaction after leaving school. However, based on the coefficient of determination, the in-school outcomes of students with disabilities could explain only a small amount of variation in their school-leaving outcomes, especially because only 2.4% of the variance was explained by their post-school employment outcomes. However, this result is inconsistent with Chiang et al. (2012) [33], who found that factors such as learning performance and parent expectations can effectively predict autistic students’ participation in postsecondary education. Chiang et al. (2013) also found that factors such as career counseling and high school vocational training programs can effectively predict employment outcomes for students with autism [34]. It may be that the participants in this study had just graduated from senior high school for one year, and the outcomes of leaving school, such as postsecondary education, employment, social adaptation, and satisfaction, had not yet been concretely manifested. Therefore, the predictive power was limited.

## 6. Conclusions

This study adopted the secondary analysis method and used data from the SNELS database to analyze the performance of the learning outcomes of students with disabilities in general classes, as well as the correlation between post-school and in-school outcomes. The results of the study found that students with disabilities had better learning performance in school, but 20% of students still had difficulties in problem-solving ability. Most parents were satisfied with the situation of students receiving education in school. On the other hand, students with disabilities performed better in postsecondary education, social adaptation, and satisfaction, and had the worst performance in employment. The impact model of learning outcomes constructed in this study shows that “school experience” had the greatest influence on the school learning outcomes model of students with disabilities, followed by student factors and family factors. Secondly, in terms of the predictive power of in-school outcomes on post-school outcomes, it was found that the in-school outcomes could effectively predict postsecondary education, employment, social adaptation, and satisfaction after leaving school, but the explanatory power of employment was weaker. 

Based on the above conclusions, this study found that school experience factors, including teacher–student relationships, peer relationships, and coursework participation, had an important impact on the learning outcomes of students with disabilities. Therefore, the researchers suggest that schools can provide peer support through regular class teachers or teacher assistants, which can effectively enhance the interaction between regular class peers and students with disabilities and the progress of IEP goals for students with disabilities [35]. It also provides regular class teachers with adjustments to teaching environments and teaching strategies [17], and support services that meet the needs of students with disabilities to improve their learning outcomes. This study also found that students with disabilities were less satisfied with the performance of their self-determination and future career development direction. The researchers also suggest that schools should strengthen the teaching of decision-making and career planning skills through relevant courses and activities, and encourage students to participate in IEP meetings and transition activities to express their own ideas and decision-making rights. This study explored the learning outcomes of students with disabilities who were placed in regular classes and its influencing factors. Due to the significant individual differences in the types and degrees of disabilities among students with disabilities, the performance of learning outcomes also varied greatly. The researchers suggest that follow-up research can explore the learning outcomes of students with different types of disabilities. In addition, this study only selected certain topics from the SNELS database as research tools. Due to the design of the database items, some measurement components had fewer topics. It is recommended that follow-up research can select more representative topics or collect longitudinal data on participants to analyze trends in learning outcomes.

## Figures and Tables

**Table 1:** Number of valid samples.

		*n*	%
Residence area	Taipei, Keelung, Yilan	124	25.0
	Taoyuan, Hsinchu, Miaoli	82	16.5
	Taichung, Changhua, Nantou	121	24.4
	Yunlin, Chiayi, Tainan	78	15.7
	Kaohsiung, Pingtung	70	14.1
	Hualien, Taitung, and Outlying Islands	21	4.2
Gender	Male	321	64.7
	Female	175	35.3
Disability category	Intellectual disabilities	16	3.2
	Visual impairments	30	6.0
	Hearing impairments	53	10.7
	Speech or language impairments	8	1.6
	Orthopedic disabilities	88	17.7
	Other health impairments	66	13.3
	Emotional and behavior disorders	46	9.3
	Learning disabilities	84	16.9
	Multiple disabilities	17	3.4
	Autism	65	13.1
	Other significant disabilities	23	4.6
	Total	496	100%

**Table 2:** Latent independent variables and observed indicators.

Latent Variables	Observed Variables	Questionnaire Source	Questions
Student	Self-concept	Student	Are you satisfied with your appearance? Do you like yourself? Do you think you have any strengths?
	Self-determination	Student	Could you solve problems by yourself? Could you make decisions by yourself?
	Learning motivation	Student	Do you feel that you learned a lot in school? Do you like going to school?
Family	Chat with kids	Parent	Do you often chat with this child?
	Parent-child relationship	Student	Will your parents talk to you about further plan? How is your relationship with your parents?
	Sibling relationship	Student	Do you get along well with your siblings? Do you often chat with your siblings?
School experience	Peer relationship	Student	How many good classmates do you have? Do you often chat with your classmates? Do you get along well with your classmates?
	Teacher-student relationship	Student	Do you like the teachers at school? Is there anyone to help when you have difficulties?
	Coursework participation	Student	Could you understand the content of the subjects? Are you interested in the content of the subjects? Will the teacher help you study?

**Table 3:** Latent dependent variables and observed indicators.

Latent Variables	Observed Variables	Questionnaire Source	Questions
In-school outcomes	Attendance and participation	Teacher	How is the student’s attendance? How does the student participate in school activities?
	Learning performance	Teacher	Pay attention in class Follow the teacher’s instructions Complete homework on time Focus on learning task
	Problem-solving ability	Teacher	Make your own choices and decisions Find a way to solve the problem Arrange your own time Understand future career development
	Parent satisfaction	Parent	The child gets along with the teachers The child gets along with classmates The child participates in school activities The child’s learning progress
Post-school outcomes	Postsecondary education	Student	Do you have enough information when choosing a university department? Do you think college life is happy? Are you satisfied with your learning performance?
	Employment	Student	Are you satisfied with the salary of the job? Do you have any difficulties at work? Do you like your current job?
	Social adaptation	Student	Could you arrange your free time? Could you do housework? Could you manage your money?
	Satisfaction	Student, Parent	Are you satisfied with your living environment? Are you happy with your life now? Are you satisfied with your child’s current living situation?

**Table 4:** Summary of in-school learning outcomes.

Latent Variables	Observed Variables	Questions	Options	Frequency	%
Learning	Attendance and participation	How is the student’s attendance?	Full attendance	227	45.8
Outcomes			Sometimes absent	260	52.4
			Often absent	7	1.4
			Almost absent	2	0.4
		How does the student participate in school activities?	All participate	348	70.2
			Mostly participated	116	23.4
			Rarely participate	25	5.0
			Never participate	7	1.4
	Learning performance	Pay attention in class	Often	248	50.0
			Sometimes	178	35.9
			Rarely	61	12.3
			Never	9	1.8
		Follow the teacher’s instructions	Often	384	77.4
			Sometimes	107	21.6
			Rarely	5	1.0
			Never	0	0.0
		Complete homework on time	Often	284	57.3
			Sometimes	154	31.0
			Rarely	50	10.1
			Never	8	1.6
		Focus on learning task	Often	243	49.0
			Sometimes	195	39.3
			Rarely	54	10.9
			Never	4	0.8
	Problem-solving ability	Make your own choices and decisions	Very agree	153	30.8
			Agree	234	47.2
			Not very agree	88	17.7
			Disagree	19	3.8
		Find a way to solve the problem	Very agree	121	24.4
			Agree	238	48.0
			Not very agree	109	22.0
			Disagree	26	5.2
		Arrange your own time	Very agree	133	26.8
			Agree	227	45.8
			Not very agree	102	20.6
			Disagree	33	6.7
		Understand future career development	Very agree	108	21.8
			Agree	211	42.5
			Not very agree	120	24.2
			Disagree	48	9.7
	Parent satisfaction	The child gets along with the teachers	Very satisfied	158	31.9
			Satisfied	285	57.5
			Not very satisfied	28	5.6
			Dissatisfied	6	1.2
		The child gets along with classmates	Very satisfied	134	27.0
			Satisfied	263	53.0
			Not very satisfied	63	12.7
			Dissatisfied	17	3.4
		The child participates in school activities	Very satisfied	121	24.4
			Satisfied	289	58.3
			Not very satisfied	44	8.9
			Dissatisfied	8	1.6
		The child’s learning progress	Very satisfied	90	18.1
			Satisfied	306	61.7
			Not very satisfied	67	13.5
			Dissatisfied	17	3.4

**Table 5:** Summary of post-school learning outcomes.

Latent Variables	Observed Variables	Questions	Options	Frequency	%
Learning	Postsecondary education	Do you have enough information when choosing a university department?	Very enough	66	16.3
Outcomes			Enough	207	51.2
			Not enough	36	8.9
			Very not enough	6	1.5
		Do you think college life is happy?	Very happy	113	28.0
			Happy	188	46.5
			Not happy	12	3.0
			Unhappy	2	0.5
		Are you satisfied with your learning performance?	Very satisfied	57	14.1
			Satisfied	207	51.2
			Not very satisfied	45	11.1
			Dissatisfied	6	1.5
	Employment	Are you satisfied with the salary of the job?	Very satisfied	7	1.7
			Satisfied	29	7.2
			Not very satisfied	6	1.5
			Dissatisfied	1	0.2
		Do you have any difficulties at work?	No difficulty	12	3.0
			Not too difficult	28	6.9
			Some difficulty	2	0.5
			Very difficult	1	0.2
		Do you like your current job?	Extremely like	8	2.0
			Slightly like	31	7.7
			Don’t really like	4	1.0
	Social adaptation	Could you arrange your free time?	Definitely yes	161	39.9
			Probably yes	176	43.6
			Probably not	59	14.6
			Definitely not	8	2.0
		Could you do housework?	Definitely yes	182	45.0
			Probably yes	165	40.8
			Probably not	44	10.9
			Definitely not	13	3.2
		Could you manage your money?	Definitely yes	191	47.3
			Probably yes	174	43.1
			Probably not	31	7.7
			Definitely not	8	2.0
	Satisfaction	Are you satisfied with your living environment?	Very satisfied	163	40.3
			Satisfied	221	54.7
			Not very satisfied	19	4.7
			Dissatisfied	1	0.2
		Are you happy with your life now?	Very happy	144	35.6
			Happy	234	57.9
			Not happy	22	5.4
			Unhappy	4	1.0
		Are you satisfied with your child’s current living situation?	Very satisfied	65	16.1
			Satisfied	269	66.6
			Not very satisfied	60	14.9
			Dissatisfied	10	2.5

**Table 6:** Summary of regression analysis of in-school outcomes and postsecondary education.

Independent Variables	B	SE_B_	*β*	R^2^	∆R^2^	F
Parent satisfaction	0.225	0.054	0.204 ***	0.054	0.054	22.826 ***
Learning performance	0.225	0.073	0.150 **	0.076	0.022	9.469 **

** *p* < 0.01 *** *p* < 0.001.

**Table 7:** Summary of regression analysis of in-school outcomes and employment.

Independent Variables	B	SE_B_	*β*	R^2^	∆R^2^	F
Learning performance	−0.198	0.063	−0.155 **	0.024	0.022	9.904 **

** *p* < 0.01.

**Table 8:** Summary of regression analysis of in-school outcomes and social adaptation.

Independent Variables	B	SE_B_	*β*	R^2^	∆R^2^	F
Parent satisfaction	0.101	0.027	0.184 ***	0.044	0.044	18.459 ***
Problem-solving ability	0.072	0.027	0.133 **	0.061	0.017	7.258 **

** *p* < 0.01 *** *p* < 0.001.

**Table 9:** Summary of regression analysis of in-school outcomes and satisfaction.

Independent Variables	B	SE_B_	*β*	R^2^	∆R^2^	F
Parent satisfaction	0.160	0.020	0.363 ***	0.132	0.132	60.742 ***

*** *p* < 0.001.

## Data Availability

The data described in this article are openly available at https://srda.sinica.edu.tw/browsingbydatatype_result.php?category=surveymethod&type=2&csid=18 (accessed on 1 March 2023).
